# Perception of the learning environment among the students in a nursing college in Eastern Nepal

**DOI:** 10.1186/s12909-019-1835-0

**Published:** 2019-10-21

**Authors:** Erina Shrestha, Ram Sharan Mehta, Gayanand Mandal, Kriti Chaudhary, Nirmala Pradhan

**Affiliations:** 1 Department of Medical Surgical Nursing, B.P. Koirala Institute of Health Sciences, Dharan, Nepal; 2 Department of Maternal Health Nursing, B.P. Koirala Institute of Health Sciences, Dharan, Nepal; 3 Department of Psychiatric Nursing, B.P. Koirala Institute of Health Sciences, Dharan, Nepal

**Keywords:** Perception, Learning environment, Students, Nursing

## Abstract

**Background:**

Learning environment is an important base for learning processes of students and for preferences of future workplaces. It is considered as an essential factor in determining the success of an effective curriculum and the students’ academic achievements. This study attempts to assess the perception of learning environment among the nursing students.

**Methods:**

A descriptive cross-sectional study design was used to conduct the study among 122 nursing students studying at B.P. Koirala Institute of Health Science. Data were collected following total enumerative sampling method using a self-administered questionnaire. Dundee Ready Educational Environment Measure (DREEM) inventory tool was used to assess the perception of learning environment. Descriptive statistics (frequency, percentage, mean and standard deviation) was used to describe the demographic and other related variables. One way Analysis of Variance (ANOVA) was used to find out the difference in the overall scale score and its subscales across the selected socio-demographic variables (age, ethnicity, residence, year of enrollment) of the respondents.

**Results:**

The mean age of the students was 21 ± 1.46 years. Majority of the students were from Province no. 1 (57.4%) and largely from Sunsari district (25.4%). First year students were found to be more satisfied (68.23%) with the educational environment (136.45 ± 16.93) compared to student of other years. Academic self-perception (21.94 ± 3.42) was the highest scoring subscale (68.57%) while the social self-perception (16.43 ± 2.96) was the lowest (58.66%). The overall DREEM score (131.25 ± 15.82 out of 200) indicated that perception of learning environment among the students was positive. Despite overall positive perception, students perceived that the teachers were authoritative and there is lack of good support system for the students at the time of stress. The total DREEM score varied significantly between the years of enrollment (*p* < 0.05).

**Conclusion:**

The current study showed positive perception of learning environment which varied significantly according to the year of enrollment. However improvements are required across all the five domains for the high quality educational environment. Future qualitative studies are recommended to confirm and to have in-depth understanding of this finding.

## Background

Educational environment is one of the most important factor in determining the success of an effective curriculum and subsequently the students’ academic success [1]. The quality of educational climate impacts the quality of the curriculum, teaching and learning consideration and developing student outcomes as practitioners [2]. Bloom described the educational or learning environment concept as “the conditions, external stimuli and forces which may be physical, social, as well as intellectual forces which challenge on the individual and influence students’ learning outcomes” [3].

A good or effective learning environment is not limited to only teacher’s good communication skills, knowledge, credibility and preparedness contributing towards teaching excellence. An environment that best prepares the students for their future professional life and contributes towards their personal and psychosomatic development as well as the social well-being is considered as an ideal academic environment [1].

As cited by Sayed and El-Sayed [4], Jiffry et al. indicated that the major domains encompassed in an educational environment of any health school are self-perception of learning, self-perception of teachers, academic self-perception, self-perception of atmosphere, and social self-perception.

Roff and McAleer have indicated that environment that is competitive, authoritarian, stressful, or threatening may de-motivate students and weaken their interest and commitment for learning process. Environment that is collaborative, collegial, and supportive may enhance greater engagement of nursing students and this may lead to improved preparedness for clinical training [4].

Previous study showed that students’ perception of their current learning environment is even a stronger predictor of learning outcomes at a university than their prior achievements at school [5]. Mayya & Roff [6] had found significant differences in the students’ perceptions of learning environment between academic achievers and under- achievers.

It is evident from recent literatures that the educational environment encountered by students has a significant impact on their behavior, satisfaction with the course of study, perceived well- being, aspirations and academic achievement [7, 8].

Studying the learning environment is important in improving the quality of an educational program [3]. The most used and accessible way of examining the educational environment is to evaluate the students’ perception of that environment [9].

Systematic review conducted by Miles et al. [8] showed that perception of the learning environment using Dundee Ready Educational Environment Measure (DREEM) has been conducted in at least 20 countries around the world. However these studies are predominantly conducted among the medical undergraduate students. Study in this regard in developing countries such as Nepal is limited, especially among nursing students.

Many methods have been tried to obtain such a reading and these include questionnaire tools, focus group studies, student feedback etc. Among them, the DREEM questionnaire is said to be one of the widely used and more specific tools in relation to assessing educational environment, especially in relation to medical education. DREEM has been validated as a universal diagnostic inventory for assessing the quality of educational environment [4, 5, 8].

Apart from this, the findings from the DREEM have been found to be consistent with qualitative information attained via interviews [10]. Secondly even though the DREEM have been used mainly for medical students, it was constructed by a panel of faculty from not only the medicals schools but also the other health professions and items were constructed based on their perceptions of learning climates conducive to education in the health profession, not just medicine [8].

## Methods

The main objective of the study was to assess the perception of learning environment among the nursing students and to find out the difference in the overall score of perception of learning environment and its subscales across the selected variables.

### Study design and setting

A descriptive cross-sectional design was employed for this study. The study was carried out among the B.Sc. Nursing students studying at B.P. Koirala Institute of Health Science (BPKIHS), tertiary level medical university in eastern Nepal.

### Participants

Required sample for this study was estimated using the formula, *n* = z^2^**σ**^2^/d^2^. Considering the study conducted by Kohli and Dhaliwal [11], where, Mean = 101.13, Standard deviation, SD (**σ**) = 21.14, Absolute precision (d) = 4.04 (4% of mean) Z_5%_ = 1.96 and adding 10% for nonresponse, the final sample size was 115. The total students currently enrolled in the program were 128; hence all the students were enrolled in the study following the total enumerative sampling method. Students who were currently enrolled in the B.Sc. Nursing program present at the time of data collection and who gave consent were included in the study.

### Instrument

Data were collected using a self-administered questionnaire based on the objectives of the research which consisted of two sections:

- Section A: socio-demographic characteristics of the students (age, ethnicity, residence and year of enrollment).
- Section B: items related to perception of learning environment based on Dundee Ready Education Environment Measure (DREEM).

DREEM is an internationally validated, non-culturally specific inventory. It includes 50 items with five point Likert scale (0–4). These items are categorized in five sub scales as Student’s Perception of Learning (12 items), Student’s Perception of Teachers (11 items), Student’s Academic Self Perception (8 items), Student’s Perception of Atmosphere (12 items), Student’s Social Self Perception (7 items). There are nine negative items (items 4, 8, 9, 17, 25, 35, 39, 48, and 50), for which correction is made by reversing the scores; thus after correction, higher scores indicate disagreement with that item. Each individual item with a mean score of ≥3.5 are true positive points; those with a mean of ≤2 are problem areas; scores in between these two limits indicate aspects of the environment that could be enhanced. The maximum global score for the questionnaire is 200 which is interpreted as follows: 0–50 = very poor, 51–100 = many problems, 101–150 = more positive than negative, 151–200 = excellent [8, 11–14]. The alpha coefficient of the tool for this study was 0.86 which indicates adequate reliability for measurement.

The research instrument was pretested to identify any ambiguities in the questionnaire. It was performed by taking 10% of the sample size i.e. 12 meeting the inclusion criteria in a different nursing college and those samples were not included in the main study.

### Ethical consideration

Ethical clearance was obtained from the Institutional Review Committee (IRC) BPKIHS and approval letter was provided by the research Committee, BPKIHS. Permission from the concerned authority was obtained to conduct the study. Permission was obtained for the use of DREEM inventory. Written informed consent was obtained from respondents prior to the data collection.

### Data collection

English version of the DREEM questionnaire was used. The questionnaire was distributed to students of each year separately at the end of the year in a leisure class. Before the questionnaire was distributed the students were briefed about the purpose of the study, data collection procedure and the meaning of some terms such as authoritarian, ridicule, factual learning which were found difficult by the students during pretesting. Researcher was present during the data collection and precautions were taken to ensure that the students didn’t copy answer from their friends’ questionnaires. Around 30 min was taken by each participant to complete the questionnaire. Total 122 students were present at the time of data collection who completed the questionnaire which were analyzed.

### Statistical analysis

The data were collected, coded, checked for completeness and entered in Microsoft EXCEL 2007 and transformed in SPSS (Statistical Package for Social Sciences) PC 11.5.0 version. Descriptive statistics (frequency, percentage, mean and standard deviation) was used to describe the demographic and other related variables. One way Analysis of Variance (ANOVA) was used to find out the difference in the score of the perception of learning environment and its subscales across the selected socio-demographic variables (age, ethnicity, residence and year of enrollment).

## Results

Around two-third (66.39%) of the respondents belonged to the age group 20–22 years. The age of the respondents ranged from 18 to 25 years with the mean age of 21 years±1.46. Around one fourth of the respondents were Brahmin (26.2%) and Mongolian (25.4%) each. Out of total students from first to fourth year, only around one fifth (19.7%) of the respondents were from second year. Students from all over Nepal were currently studying in the B.Sc. nursing program. More than half (57.4%) of the respondents were from Province no. 1. Around one fourth (25.4%) of the respondents were from Sunsari district. Almost half (47.6%) of the respondents were from the other 28 districts altogether. As BPKIHS is located in Sunsari district of Province no. 1, the students near to this area may be more interested in studying here.

The overall score for perception of learning environment was 131.25 ± 15.82 (65.62% of maximum score). Among the five subscales, Student’s Academic self-perception was the highest scoring subscale (68.57%) with the mean score of 21.94 ± 3.42 while the social self-perception was the lowest (58.66%) with the mean score of 16.43 ± 2.96. Subscales means and standard deviation along with percentage are depicted in Table 3.

On individual item analysis, the mean of individual items revealed that ten items scored three or more than three which indicated the positive aspect. However none of the items scored more than 3.5. Scores of six items were below two. The mean scores of the majority of the items were between two and three.

Significant differences were found between the years of enrollment for the overall perception of the learning environment (*p* < 0.05). The score for subscale student’s perception of teachers also varied significantly between the years of enrollment (*p* < 0.05). There was no significant difference in the score of the perception of the learning across the other selected variables. The details of the result are depicted in the Tables 1, 2, 3 and 4.

**Table 1:** Socio-demographic Characteristics of the Respondents (*n* = 122)

Characteristics	Category	Frequency	Percentage (%)
Age (completed years)	≤19	19	15.57
	20–22	81	66.39
	> 22	22	18.04
Mean ± SD: 21 ± 1.46, Range (18–25)			
Ethnicity	Brahmin	32	26.2
	Chhetri	18	14.8
	Newar	10	8.2
	Mongolian	31	25.4
	Terai origin	23	18.9
	Others	8	6.6
Class	First Year	40	32.8
	Second Year	24	19.7
	Third Year	30	24.6
	Fourth Year	28	23.0
Residence (Province)	1	70	57.4
	2	23	18.9
	3	12	9.8
	4	9	7.4
	5	4	3.3
	6	1	0.8
	7	3	2.5
Residence (District)	Sunsari	31	25.4
	Morang	21	17.2
	Jhapa	12	9.8
	Others (28 districts)	58	47.6

**Table 2:** Mean scores of individual items of Dundee Ready Educational Environment Measure (DREEM)

SN	Domain Items	Mean	SD
Students’ Perception of Learning			
1.	I am encouraged to participate in class	3.01	0.77
2.	The teaching is often stimulating	2.58	0.78
3.	The teaching is student centered	2.60	0.86
4.	The teaching helps to develop my competence	3.18	0.70
5.	The teaching is well focused	2.72	0.79
6.	The teaching helps to develop my confidence	3.19	0.60
7.	The teaching time is put to good use	2.79	0.79
8.	The teaching over-emphasizes factual learning ^a^	1.49	0.80
9.	I am clear about the learning objectives of the course	2.90	0.73
10.	The teaching encourages me to be an active learner	2.88	0.75
11.	Long term learning is emphasized over short term learning	2.70	0.84
12.	The teaching is too teacher-centered ^a^	2.19	1.03
Students’ Perception of Teachers			
13.	The teachers are knowledgeable	3.21	0.62
14.	The teachers are patient with patients	2.84	0.72
15.	The teachers ridicule the students ^a^	2.41	0.95
16.	The teachers are authoritarian ^a^	1.37	0.95
17.	The teachers have good communication skills with patients	2.99	0.79
18.	The teachers are good at providing feedback to students	3.05	0.80
19.	The teachers provide constructive criticism here	2.39	0.89
20.	The teachers give clear examples	2.71	0.81
21.	The teachers get angry in class ^a^	2.30	1.02
22.	The teachers are well prepared for their classes	2.75	0.85
23.	The students irritate the teachers ^a^	2.85	0.88
Students’ Academic Self-perception			
24.	Learning strategies which worked for me before continue to work for me now	2.31	1.03
25.	I am confident about my passing this year	3.11	0.73
26.	I feel I am being well prepared for my profession	2.91	0.75
27.	Last year’s work has been a good preparation for this year’s work	2.57	0.89
28.	I am able to memorize all I need	1.93	0.92
29.	I have learned a lot about empathy in my profession	3.21	0.71
30.	My problem solving skills are being well developed here	2.84	0.71
31.	Much of what I have to learn seems relevant to a career in healthcare	3.06	0.60
Students’ Perception of Atmosphere			
32.	The atmosphere is relaxed during the ward teaching	2.02	1.05
33.	This school is well timetabled	2.16	1.32
34.	Cheating is a problem in this school ^a^	2.70	1.37
35.	The atmosphere is relaxed during lectures	2.80	0.74
36.	There are opportunities for me to develop interpersonal skills	3.30	0.64
37.	I feel comfortable in class socially	3.02	0.60
38.	The atmosphere is relaxed during seminars/tutorials	2.92	0.75
39.	I find the experience disappointing ^a^	2.40	1.04
40.	I am able to concentrate well	2.48	0.86
41.	The enjoyment outweighs the stress of the course	2.50	0.95
42.	The atmosphere motivates me as a learner	2.70	0.79
43.	I feel able to ask the questions I want	2.78	0.91
Students’ Social Self-perception			
44.	There is a good support system for students who get stressed	1.69	1.17
45.	I am too tired to enjoy the course ^a^	1.98	1.09
46.	I am rarely bored on this course	1.90	1.07
47.	I have good friends in this school	3.20	0.77
48.	My social life is good	2.89	0.68
49.	I seldom feel lonely	2.10	1.20
50.	My accommodation is pleasant	2.67	0.85

^a^Reverse scoring

**Table 3:** Scores of Overall Perception of Learning Environment and its subscales (*n* = 122)

Characteristics	No. of items	Obtainable score	Mean	SD	Mean Percent
Students’ Perception of Learning	12	0–48	32.22	4.49	67.12
Students’ Perception of Teachers	11	0–44	28.89	4.39	65.64
Students’ Academic Self-perception	8	0–32	21.94	3.42	68.57
Students’ Perception of Atmosphere	12	0–48	31.78	4.94	66.20
Students’ Social Self-perception	7	0–28	16.43	2.96	58.66
Total Perception of Learning Environment	50	0–200	131.25	15.82	65.62

**Table 4:** Association of Perception of learning environment and its subscales with year of enrollment (*n* = 122)

Domains	First Year	Second Year	Third Year	Fourth Year	*P*- value ^a^
Students’ Perception of Learning	33.38 ± 4.57	32.42 ± 4.56	32.23 ± 4.64	30.39 ± 3.72	0.61
Students’ Perception of Teachers	30.58 ± 4.99	28.21 ± 3.52	28.70 ± 4.30	27.25 ± 3.52	0.01
Students’ Academic Self-perception	22.35 ± 3.62	21.33 ± 3.27	22.43 ± 3.72	21.36 ± 2.89	0.43
Students’ Perception of Atmosphere	32.98 ± 5.55	31.00 ± 4.72	31.83 ± 4.67	30.68 ± 4.28	0.22
Students’ Social Self-perception	17.18 ± 3.15	16.83 ± 3.14	15.93 ± 2.75	15.54 ± 2.50	0.09
Total Perception of Learning Environment	136.45 ± 16.93	129.79 ± 15.89	131.13 ± 16.37	125.21 ± 11.19	0.03

^a^One way ANOVA

## Discussion

The overall DREEM mean score obtained in this study indicated that the student’s perception of learning environment was positive. Though this score highlighted the student centered approach followed in the nursing program, improvements are needed to have a positive impact on the student’s achievement, satisfaction and success [15]. Study conducted by Roff et al. [12] in Nepalese Health Profession Institution showed similar overall DREEM score of 130. Similar to this study, positive perception of learning environment was seen in several other studies conducted in various Nursing colleges of Malaysia [3], Suadi Arabia [4] and Iran [9]. Similar finding was seen in studies conducted by Arab [16], Imanipour [17], Bakshi [18] and Victor [19] that showed the overall DREEM score of 103.54, 104.39, 114.3 and 119 respectively. A study conducted among medical and nursing undergraduate students of Srilanka also showed positive perception [20]. Similar studies were predominantly conducted in medical schools of India [1, 11], Pakistan [21], Malaysia [22], Saudi Arabia [23], Iran [24], Egypt [25], Australia [15], Brazil [26], Sweden [14] and the findings from those studies were in agreement with that of the current study.

In contrast to the present finding, a study conducted in Egypt [7] among the nursing students showed poor perception towards their learning environment. Contrast result to the present finding was also seen in study conducted by Al-Ayed [27] in a medical college in Riyadh with the overall DREEM score of 89.9. Studies conducted by Aghamolaei [28] and Taheri [2] in medical schools of Iran also reported potential problems with the total DREEM score of 99.6, 98 respectively. However no studies were found by the researcher in which the perception of the learning environment was excellent. Variation in population and setting studied might be the reason for variation in score.

In the present study, the interpretation of each five subscales of DREEM revealed a perception which was directed more towards the positive side. Similar finding was seen in the study conducted by Said [3], Sayed [4], Mayya [6], Farajpour [9], Bakshi [24] and Sajid [29].

On individual item analysis ten items scored three or more than three which indicated the positive aspect. The students perceived that there are opportunities to develop their interpersonal skills, the teachers are knowledgeable and good at providing feedback, the teaching helped to develop their competence and confidence. The students are confident about passing the exam. They have good friends and are comfortable socially. These all findings revealed the liberal atmosphere present in the institution.

According to Mayya and Roff [6], items scoring more than 3.5 are the excellent areas. However in the present study none of the items scored more than 3.5 which indicated that there were no particularly excellent areas and lot of areas needed improvement. This finding is consistent with the findings of the study conducted by Abussad [30].

Six items scored two or less than two. These low scores are the areas of concern. The student identified that increased tiredness and stress to enjoy the course, lack of good stress support system, factual learning and authoritarian teachers as significant problems. The impression that the teachers are authoritarian and emphasized on factual learning has also been shown by other studies conducted in India [1] and Iran [5]. Perceived lack of good support system is also seen in a study conducted in Iran by Aghamolaei [28] and Imanipour [17]. For a positive academic environment, the overall well-being of the students’ needs to be taken into consideration in terms of workload maintaining a balance between the academic activities and the recreation time [1] so that they can enjoy the course. The findings from the present study indicate that the support system provided by the faculty and the institution should be improved in order to facilitate the learning of the students. As most of the students are not locals and need to stay away from their parents and guardians, the students should be made aware of the available support system such as emphasizing the role of the preceptors who are accessible in the institution to help the students. It also focuses on to improvise the student centered approach which is followed in the nursing program.

Significant difference was found between the perception of learning environment and the year of enrollment. This finding is in accordance to the findings given by Roff [12], Said [3], Brown [15], Bakshi [18] and Bakshi [24]. However no any significant difference was found between the perception of learning environment and the other selected socio-demographic variables.

Out of total students, first year students had the highest mean score for the perception followed by third year students, second year students and the fourth year students respectively. This trend was fairly consistent among the different subscales where first year students scored more than the other years. This might be explained by the fact that first year students are not exposed to all the areas and are not too stressed by the study. The positive perception among the newcomers might have been fueled by the excitement of getting admissions in one of the reputed institution of the nation. A study conducted by Said [3] in Malaysia among the nursing students also revealed highest DREEM score among the first year students. Similar finding was seen in a study conducted by Bakshi [18] and Al-Ayed [27] among the nursing students in Iran and Suadi Arabia. However a study conducted by Roff et al. [12] in Nepalese Health profession institutions showed improved perception in the year 2 and 3 over year 1 as reflected by the DREEM scores across the years. Study conducted by Bakshi [24] in a medical college in Iran also revealed higher scores for the year second and fourth.

This study provides the valuable insight regarding the educational environment as perceived by the nursing students. The study is conducted in a single institution among limited participants. So it would be important to conduct the similar studies in other nursing institutions across the country for establishing the generalizability of the findings. Qualitative studies are recommended to have in-depth understanding of the findings, address the specific problems and highlight the strength of the particular learning environment.

## Conclusion

In conclusion students in this institution revealed a positive perception of the learning environment, which varied significantly according to the year of enrollment. However improvements are required across all the five domains for the high quality educational environment.

## Data Availability

The datasets generated and/or analysed during the current study are available from the corresponding author on reasonable request.

## Abbreviations

ANOVA: One Way Analysis of Variance
BPKIHS: B. P. Koirala Institute of Health Science
DREEM: Dundee Ready Educational Environment Measure
IRC: Institutional Review Committee
SD: Standard Deviation
SPSS: Statistical Package for Social Sciences
